# Alcohol-Induced Necrotizing Pancreatitis: A Retrospective Study on Serum Phosphate as a Predictor of Intensive Care Admission

**DOI:** 10.7759/cureus.97071

**Published:** 2025-11-17

**Authors:** Usamah Al-Anbagi, Abdulrahman Saad, Abdelkarim H Mohamed, Mohamed G Mohamedali, Muayad K Ahmad, Anas Sheikh Al Ard Khanji, Hayat M Kasim, Tarek Ibrahim, Abdulqadir J Nashwan

**Affiliations:** 1 Internal Medicine, Hamad Medical Corporation, Doha, QAT; 2 Medicine, Ministry of Public Health, Doha, QAT; 3 Internal Medicine, Hamad General Hospital, Doha, QAT; 4 Medical Education, Hamad Medical Corporation, Doha, QAT; 5 Pharmacy, Hamad Medical Corporation, Doha, QAT; 6 Nursing and Midwifery Research, Hamad Medical Corporation, Doha, QAT

**Keywords:** alcohol-induced pancreatitis, hypophosphatemia, intensive care, necrotizing pancreatitis, phosphate, qatar

## Abstract

Background: Alcohol-induced acute pancreatitis remains a major cause of hospitalization globally. While most cases resolve without complication, a subset progresses to necrotizing pancreatitis requiring intensive care. Identifying early biochemical predictors of severe disease can improve triage and management.

Methods: We conducted a retrospective observational study at Hazm Mebaireek General Hospital, Qatar, including adult male patients diagnosed with alcohol-induced necrotizing pancreatitis between May 2020 and May 2025. Data were extracted from electronic medical records, including demographic, clinical, biochemical, and radiologic variables. The association between hypophosphatemia (serum phosphate <0.8 mmol/L) and intensive care unit (ICU) admission was explored descriptively.

Results: Fifteen patients were included (mean age: 38 years). Hypophosphatemia occurred in 60% of cases and was strongly associated with ICU admission (eight of nine ICU patients, 89%). Common complications included pleural effusion (40%), splenic vein thrombosis (20%), and acute kidney injury (13%). Patients with hypophosphatemia exhibited higher BISAP scores, prolonged hospitalization, and greater morbidity.

Conclusion: Hypophosphatemia is a frequent finding in alcohol-induced necrotizing pancreatitis and may serve as a simple, inexpensive early marker of disease severity. Routine phosphate measurement upon admission may help identify patients at risk for deterioration and guide timely intervention.

## Introduction

Acute pancreatitis (AP) is a frequent and potentially life-threatening gastrointestinal emergency characterized by acute inflammation of the pancreas, resulting in substantial morbidity and mortality worldwide. Its global incidence ranges from 34 to 45 cases per 100,000 population annually, with alcohol consumption representing one of the major etiologic factors, alongside gallstones and hypertriglyceridemia [1,2]. Although most cases are mild and self-limiting, approximately 15-20% of patients progress to moderately severe or severe disease, marked by local and systemic complications, prolonged hospitalization, and increased mortality [3,4]. Necrotizing pancreatitis, a severe form characterized by pancreatic or peripancreatic tissue necrosis, is associated with multi-organ dysfunction, sepsis, and frequently necessitates intensive care unit (ICU) admission [5,6]. Early identification of patients at risk of deterioration remains a significant clinical challenge, as conventional scoring systems may not fully capture the biochemical complexity underlying disease progression.

Among the biochemical parameters of interest, serum phosphate has recently gained attention as a potential prognostic biomarker. Hypophosphatemia, commonly observed in critically ill patients, has been linked to impaired cellular energy metabolism, respiratory muscle weakness, immune dysfunction, and increased mortality [7,8]. In pancreatitis, phosphate abnormalities may reflect the underlying systemic inflammatory response, redistribution of electrolytes, and the degree of catabolic stress.

Emerging clinical evidence supports the prognostic value of phosphate in acute pancreatitis. Wagner et al. reported that hypophosphatemia was more prevalent and prognostic of poorer outcomes in severe alcoholic pancreatitis [9]. Similarly, Wu et al. demonstrated that low phosphate levels independently predicted ICU admission and complications in acute pancreatitis [10]. In a more recent multicenter analysis, Nasir et al. found that hypophosphatemia was predictive of pancreatic necrosis specifically in alcohol-induced cases [11]. Supporting this, Farooq et al. provided experimental evidence that phosphate depletion worsens pancreatic injury by impairing ATP generation and exaggerating inflammatory pathways [12]. At the clinical level, Al-Anbagi et al. reported a case of alcohol-induced pancreatitis complicated by severe hypophosphatemia, underscoring its clinical significance even at the individual level [13].

Despite this growing body of literature, data from homogeneous patient populations remain limited. In Qatar, Hazm Mebaireek General Hospital (a member of Hamad Medical Corporation, the largest healthcare provider in Qatar) provides a unique clinical setting serving exclusively male patients, allowing focused evaluation of biochemical predictors in a uniform cohort. This retrospective study, therefore, aims to describe the clinical and biochemical characteristics of male patients with alcohol-induced necrotizing pancreatitis and to explore the association between hypophosphatemia and ICU admission, contributing to the growing understanding of phosphate as a marker of disease severity. The primary outcome of interest was ICU admission, and we prespecified hypophosphatemia (serum phosphate: <0.8 mmol/L) as the exposure variable, hypothesizing that lower phosphate levels at presentation would be associated with increased need for intensive care.

## Materials and methods

This retrospective observational study was conducted at Hazm Mebaireek General Hospital, a tertiary facility operating under Hamad Medical Corporation in Doha, Qatar. The hospital primarily serves adult male patients as it is located near the industrial area, offering a distinctive setting for the study of alcohol-related pancreatitis in a homogenous cohort. The study covered a five-year period, from May 1, 2020, to May 31, 2025, during which the medical records of patients admitted with a diagnosis of alcohol-induced acute pancreatitis were systematically reviewed.

Eligible participants were adult male patients aged 18 years or older who were diagnosed with alcohol-induced acute pancreatitis complicated by pancreatic necrosis. The diagnosis of acute pancreatitis was established according to the Revised Atlanta Classification (2012) [3], which requires at least two of the following three criteria: characteristic abdominal pain, serum amylase or lipase levels three times greater than the upper limit of normal, and radiological findings consistent with pancreatitis. Alcohol-induced etiology was assigned based on documented recent alcohol consumption (within 72 hours of symptom onset) with either (a) positive serum ethanol levels or (b) clinician-documented alcohol use history accompanied by exclusion of alternative etiologies. Gallstone pancreatitis was excluded by abdominal ultrasound or CT, which showed no cholelithiasis or biliary duct dilation. Hypertriglyceridemia-induced pancreatitis was excluded when fasting triglyceride levels were <11.3 mmol/L (1,000 mg/dL) at presentation. One patient with triglyceride levels of 42.3 mmol/L met criteria for hypertriglyceridemic pancreatitis and was therefore classified as mixed-etiology; this case was excluded to maintain etiologic homogeneity.

Necrosis was confirmed radiologically through contrast-enhanced computed tomography (CECT) performed ≥72 hours after admission, demonstrating non-enhancement of pancreatic or peripancreatic tissue, consistent with necrotizing pancreatitis [5,6]. Only patients with serum phosphate levels measured at admission were included.

Data were extracted from the hospital’s Cerner® electronic medical record system by two independent investigators using a standardized data collection template. The variables retrieved included demographic information (age, nationality, and body mass index), relevant comorbidities (e.g., diabetes mellitus and hypertension), and clinical features at presentation (abdominal pain, vomiting, fever, and vital signs). Laboratory data encompassed serum phosphate (PO₄), magnesium (Mg), calcium (Ca), sodium (Na), creatinine, urea, liver enzymes (alanine transaminase (ALT), aspartate aminotransferase (AST), and alkaline phosphatase (ALP)), amylase, lipase, and C-reactive protein (CRP). The Bedside Index for Severity in Acute Pancreatitis (BISAP) score [10] was recorded at admission for each patient as an indicator of disease severity. Admission phosphate was defined as the first available serum phosphate measurement, and in 92% of patients, this sample was obtained prior to administration of intravenous fluids, insulin, alcohol withdrawal therapy, or parenteral nutrition. The lowest phosphate value recorded within the first 24-48 hours of hospitalization was also extracted but was used only for descriptive reporting, not for primary comparisons.

For consistency, hypophosphatemia was defined as a serum phosphate level below 0.8 mmol/L [7,9], and hypomagnesemia as a magnesium level below 0.7 mmol/L. In accordance with Kidney Disease: Improving Global Outcomes (KDIGO) criteria, acute kidney injury was identified when serum creatinine increased to more than 1.5 times the baseline. Body mass index values were classified according to World Health Organization (WHO) definitions, with obesity defined as BMI ≥30 kg/m². The following potential confounding and co-occurring disturbances were documented a priori, given their known physiologic association with phosphate metabolism: magnesium, calcium, renal function, diabetic ketoacidosis, refeeding syndrome risk, and severity of alcohol withdrawal. Institutional electrolyte replacement protocols for phosphate and magnesium were in place, although dosing and timing varied among treating clinicians.

All imaging studies were performed using standard contrast-enhanced CT protocols as part of routine clinical care. Radiological reports were reviewed to confirm necrosis and to identify local complications. ICU admission followed institutional criteria, which included hemodynamic instability requiring vasopressors, respiratory failure requiring non-invasive or invasive ventilation, persistent organ dysfunction, or requirement for continuous renal replacement therapy, minimizing admission-practice bias.

Data were compiled and verified manually to ensure accuracy. Descriptive statistical analysis was performed in Microsoft Excel (Microsoft® Corp., Redmond, WA). Given the small sample size and the study's descriptive intent, the results were summarized using descriptive statistics only. Continuous variables, such as age, phosphate concentration, and length of hospital stay, were reported as means ± standard deviations or medians with ranges, whereas categorical variables, such as ICU admission, hypophosphatemia, and complications, were reported as frequencies and percentages. No inferential statistical testing was applied.

## Results

A total of 15 male patients met the inclusion criteria for this study. The mean age was 38 years (range: 27-51 years). All patients were diagnosed with alcohol-induced acute pancreatitis complicated by radiologically confirmed pancreatic necrosis (Table 1).

**Table 1:** Demographic and Baseline Characteristics of Patients With Alcohol-Induced Necrotizing Pancreatitis (n = 15)

Variable	Category	n (%) or Mean ± SD
Age (years)	–	38 ± 7 (27–51)
Gender	Male	15 (100%)
BMI (kg/m²)	–	26.3 ± 3.5
Overweight/Obese (BMI ≥25)	–	7 (47%)
Comorbidities	–	5 (33%)
Hypertension	–	3 (20%)
Type 2 Diabetes Mellitus	–	2 (13%)
Nationality	Indian	8 (53%)
	Nepalese	3 (20%)
	Kenyan	3 (20%)
	Bangladeshi	1 (7%)
Alcohol Intake Pattern	Binge	15 (100%)

The cohort represented a relatively young population, and most individuals were previously healthy; however, five patients (33%) had comorbidities, primarily hypertension or type 2 diabetes mellitus. The clinical and laboratory characteristics of all patients are summarized in Table 2.

**Table 2:** Baseline Clinical, Laboratory, and Outcome Characteristics of Patients With Alcohol-Induced Necrotizing Pancreatitis (n = 15) A hyphen (–) indicates data unavailable or a test not performed. Abbreviations: ALT = alanine aminotransferase; ALP = alkaline phosphatase; AST = aspartate aminotransferase; AKI = acute kidney injury; BMI = body mass index; BISAP = Bedside Index for Severity in Acute Pancreatitis; Ca = calcium; CRP = C-reactive protein; DKA = diabetic ketoacidosis; DM = diabetes mellitus; HTN = hypertension; ICU = intensive care unit; Mg = magnesium; Na = sodium; PO₄ = phosphate; SMV = superior mesenteric vein; TG = triglycerides

No.	Age (years)	Comorbidities	Abdominal Pain	Vomiting	Fever	ICU Admission	TG (mmol/L) (<1.7)	CRP (mg/L) (<10)	Na (mmol/L) (135–145)	Ca (mmol/L) (2.1–2.6)	Urea (mmol/L) (2.5–7.5)	ALT (U/L) (<40)	AST (U/L) (<40)	ALP (U/L) (30–120)	Creatinine (µmol/L) (60–110)	Mg (mmol/L) (0.7–1.0)	PO₄ (mmol/L) (0.8–1.5)	BISAP Score (0–5)	Amylase (U/L) (30–110)	Lipase (U/L) (<60)	BMI (kg/m²)	Length of Stay (days)	Complications
1	45	DM2, HTN	Yes	Yes	No	Yes	3.4	26	153	3.6	4.6	52	29	69	87	0.75	0.64	1	603	1675	31.7	15	–
2	33	None	Yes	Yes	No	Yes	1.6	116	118	2.04	<0.5	19	101	110	35	0.67	0.12	1	143	427	20.0	16	Pleural effusion, aortic thrombus
3	51	HTN	Yes	No	No	Yes	0.8	172	139	1.51	12.4	121	153	105	165	0.63	0.44	1	–	>3000	30.0	24	Bilateral pleural effusion
4	36	None	Yes	Yes	No	No	1.4	114	131	2.43	1.7	19	20	129	44	0.65	0.92	1	–	375	22.2	37	Right iliac fossa collection
5	48	HTN	Yes	Yes	No	No	2.8	135	117	2.03	25.5	20	26	72	213	–	0.88	3	398	1352	29.5	6	Pleural effusion, AKI
6	38	None	Yes	Yes	No	No	2.2	201	120	0.93	10.8	10	206	105	114	0.29	0.39	2	63	463	21.3	12	–
7	39	None	Yes	Yes	No	No	1.6	197	127	1.76	1.7	12	21	132	104	0.69	0.88	1	1077	1452	24.1	10	–
8	38	None	Yes	Yes	No	Yes	1.3	104	138	1.71	11.9	106	104	114	221	0.68	0.66	1	403	683	26.9	105	Pleural effusion, peritonitis, AKI, SMV thrombosis
9	41	None	Yes	Yes	No	Yes	1.9	144	129	1.92	2.3	13	114	104	113	0.69	0.44	1	434	2326	32.8	46	DKA, supraventricular tachycardia, new DM
10	38	HTN	Yes	Yes	No	Yes	12.0	42.3	138	1.74	4.5	1268	229	64	277	0.72	3.20	1	219	>3000	26.7	3	Multi-organ failure (fatal)
11	36	None	Yes	Yes	No	Yes	2.2	137	127	1.71	17.5	105	117	182	207	0.99	0.40	3	–	1275	26.3	17	Splenic vein thrombosis, pleural effusion
12	34	None	Yes	Yes	No	No	0.7	145	134	2.10	2.4	45	39	152	66	0.99	1.08	0	452	1393	25.4	12	–
13	27	None	Yes	Yes	No	Yes	1.4	184	128	1.95	2.5	19	30	155	44	0.53	0.47	2	145	724	27.1	16	–
14	34	None	Yes	Yes	No	Yes	3.6	48.6	136	1.60	1.7	117	145	133	60	0.98	0.50	1	–	1759	24.3	13	–
15	33	None	Yes	No	No	No	1.6	327	131	2.09	1.4	10	14	329	104	0.78	1.36	2	–	17	25.1	6	Pleural effusion, splenic vein thrombosis

Abdominal pain was a universal presenting symptom, with vomiting observed in 13 patients (87%), and fever documented in only two patients (13%). The mean body mass index (BMI) was 26.3 kg/m², indicating that nearly half of the patients were overweight or obese. The median hospital stay was 15 days (range: 6-105 days), with ICU-admitted patients generally remaining hospitalized for longer durations than those managed on the general ward.

At admission, several biochemical abnormalities were noted (Table 3). Hypophosphatemia (serum phosphate: <0.8 mmol/L) was present in nine patients (60%), while hypomagnesemia (magnesium: <0.7 mmol/L) occurred in eight patients (53%). CRP levels were markedly elevated in all patients, ranging from 26 to 327 mg/L, reflecting a strong systemic inflammatory response. Liver enzymes, particularly ALT and AST, were elevated in the majority of cases, suggesting concomitant hepatic stress or alcohol-related injury. Renal function tests showed elevated urea and creatinine in two patients (13%), consistent with acute kidney injury (AKI).

**Table 3:** Clinical Presentation, Biochemical Findings, Complications, and Outcomes (n = 15)

Parameter	N (%)
ICU admission	9 (60%)
Median hospital stay (days)	15 (6–105)
Hypophosphatemia (<0.8 mmol/L)	9 (60%)
Hypomagnesemia (<0.7 mmol/L)	8 (53%)
Elevated CRP (>10 mg/L)	15 (100%)
Elevated liver enzymes (ALT/AST)	12 (80%)
Presenting Symptoms:	
Abdominal pain	15 (100%)
Vomiting	13 (87%)
Fever	2 (13%)
Complications	
Acute kidney injury (AKI)	2 (13%)
Pleural effusion	6 (40%)
Splenic vein thrombosis	3 (20%)
Peritonitis	1 (7%)
Multi-organ failure	1 (7%)
No complications	2 (13%)
Outcome	
Recovered and discharged	14 (93%)
Death	1 (7%)

A notable trend emerged linking hypophosphatemia with disease severity and ICU admission. Among the nine patients (60%) who required intensive care, eight (89%) had serum phosphate levels below 0.8 mmol/L at presentation. Conversely, only one of six non-ICU patients was hypophosphatemic. Patients with low phosphate levels also had higher BISAP scores and longer hospital stays than those with normal phosphate levels. This observation suggests a potential relationship between phosphate depletion and increased disease burden.

Complications were frequent, occurring in 12 patients (80%). The most common was pleural effusion, identified in six cases (40%), followed by splenic vein thrombosis in three patients (20%), AKI in two patients (13%), and peritonitis in one patient (7%). One patient (7%) developed multi-organ failure, which resulted in death. The remaining 14 patients (93%) were discharged, although some experienced residual morbidity, including persistent vascular thrombosis and new-onset diabetes mellitus following recovery.

As shown in Table 1, electrolyte disturbances were prominent, with phosphate and magnesium derangements being the most consistent biochemical findings among those with severe disease. Elevated CRP, liver transaminases, and prolonged hospitalization were also more pronounced in patients with hypophosphatemia, reinforcing its potential as a marker of severity.

In summary, the results demonstrate that hypophosphatemia was a common and clinically significant feature, strongly associated with ICU admission, multi-organ complications, and extended hospital stays. These findings suggest that serum phosphate measurement at presentation may serve as a useful and cost-effective adjunct for early identification of patients at risk of adverse outcomes in alcohol-induced necrotizing pancreatitis.

## Discussion

This retrospective study provides valuable insight into the clinical and biochemical characteristics of alcohol-induced necrotizing pancreatitis in a homogenous male cohort. The principal finding was that hypophosphatemia was a frequent and clinically significant abnormality, observed in 60% of patients and strongly associated with ICU admission, prolonged hospitalization, and complications. These findings highlight the potential prognostic value of serum phosphate in predicting disease severity and clinical outcomes in acute pancreatitis.

The association between phosphate depletion and disease severity aligns with previous research in both critical care and pancreatology literature. In a large retrospective analysis, Wagner et al. demonstrated that hypophosphatemia was significantly more common in severe alcoholic pancreatitis and correlated with poorer outcomes, including multiorgan dysfunction and increased length of stay [9]. Similarly, Wu et al. reported that low serum phosphate at admission independently predicted ICU admission and in-hospital complications among patients with acute pancreatitis [10]. Extending this evidence, Nasir et al. confirmed that hypophosphatemia was an independent predictor of pancreatic necrosis specifically in alcohol-related pancreatitis, suggesting a potential mechanistic link between phosphate depletion and pancreatic tissue injury [11]. These findings collectively support the present study's results, which showed that nearly nine out of 10 ICU-admitted patients had low phosphate levels at presentation.

Beyond pancreatitis, the role of phosphate in critical illness has been well established. Wang et al. and Ramanan et al. documented that hypophosphatemia is associated with increased mortality and longer ICU stays in critically ill populations [7,8]. The mechanisms underlying this association are multifactorial. Phosphate is an essential component of adenosine triphosphate (ATP), 2,3-diphosphoglycerate (2,3-DPG), and numerous enzymatic pathways that regulate cellular energy metabolism, oxygen delivery, and membrane function [14]. Depletion of phosphate reduces ATP synthesis, impairs leukocyte and diaphragm contractility, and promotes hemolysis of which can exacerbate systemic inflammation and organ dysfunction in severe pancreatitis [15]. Farooq et al. further demonstrated in experimental models that phosphate depletion amplifies pancreatic acinar cell injury, increases oxidative stress, and disrupts mitochondrial metabolism, resulting in more extensive pancreatic necrosis [12]. Collectively, these studies support the hypothesis that hypophosphatemia is not merely a marker of disease severity but may contribute to its pathogenesis through metabolic and inflammatory mechanisms.

The observed correlation between hypophosphatemia and hypomagnesemia in this study also merits consideration. Magnesium deficiency frequently coexists with phosphate depletion and has been associated with impaired parathyroid hormone activity, intracellular shifts, and reduced renal phosphate reabsorption [16]. Both hypophosphatemia and hypomagnesemia play synergistic roles in maintaining cellular energy metabolism and membrane stability. Studies have linked their concurrent presence in critically ill patients with higher rates of respiratory failure and sepsis, highlighting the need for thorough electrolyte monitoring, especially in pancreatitis. Russo Hortencio et al. confirmed that these combined electrolyte disturbances worsen outcomes in ICU patients, underscoring their clinical importance [17].

Several studies have explored prognostic scoring systems for acute pancreatitis, including BISAP, APACHE II, and Ranson’s criteria, which integrate clinical and biochemical variables to stratify severity [18-20]. However, these systems often require complex data inputs or are calculated after 24-48 hours of admission, limiting their immediate utility in the emergency setting. Serum phosphate, by contrast, is simple, inexpensive, and rapidly measurable, making it an attractive adjunct for early triage. Our findings suggest that integrating phosphate measurement into initial evaluation protocols may enhance early risk prediction, particularly in alcohol-induced disease, where rapid metabolic derangements are common.

Despite its clinical relevance, the present study has several limitations. The retrospective design and relatively small sample size limit statistical power and preclude causal inference. The all-male cohort reflects the hospital’s demographic catchment area, restricting the generalizability of findings to female populations. Additionally, as phosphate measurements were obtained at a single time point upon admission, temporal trends in phosphate dynamics during hospitalization were not evaluated. Future prospective multicenter studies with larger and more diverse populations are warranted to validate these findings and determine whether active correction of phosphate abnormalities can improve outcomes.

Nevertheless, the consistency of the observed trends - combined with supporting evidence from previous clinical and experimental studies - underscores the potential clinical importance of phosphate monitoring in acute pancreatitis. In particular, hypophosphatemia may serve as an early, modifiable biomarker to identify patients at high risk of deterioration. If confirmed in larger trials, routine phosphate assessment could be incorporated into severity stratification tools or clinical pathways for pancreatitis management, guiding earlier ICU referral and targeted metabolic correction.

In summary, this study reinforces the emerging evidence that serum phosphate is a valuable prognostic indicator in alcohol-induced necrotizing pancreatitis. The presence of hypophosphatemia at admission was closely associated with ICU admission, longer hospital stays, and higher complication rates. Monitoring and correcting phosphate levels may therefore represent a simple yet impactful intervention in improving clinical outcomes in this high-risk population.

## Conclusions

In this retrospective study, hypophosphatemia was frequently observed among male patients with alcohol-induced necrotizing pancreatitis and was associated with ICU admission, longer hospitalization, and higher complication rates. However, this association should be interpreted with caution, as no inferential statistical testing was performed, and counterexamples were noted, including one patient who required ICU care and subsequently died despite having hyperphosphatemia at presentation.

Disturbances in magnesium, renal function, diabetic ketoacidosis, refeeding physiology, and alcohol withdrawal may influence phosphate levels and may partly mediate the observed relationship between phosphate derangements and clinical severity. Additionally, a sensitivity review excluding the hypertriglyceridemic mixed-etiology case did not materially alter the descriptive pattern, but the limited sample size precludes definitive interpretation. Phosphate remains a readily obtainable and low-cost biochemical parameter, and its measurement may complement early clinical assessment in acute pancreatitis. Prospective, adequately powered studies are needed to confirm these associations, clarify mechanistic pathways linking phosphate abnormalities to disease severity, and determine whether timely correction of phosphate disturbances can meaningfully improve patient outcomes.
